# Assessing Information Available for Health Professionals and Potential Participants on Lung Cancer Screening Program Websites: Cross-sectional Study

**DOI:** 10.2196/34264

**Published:** 2022-08-30

**Authors:** Rachael H Dodd, Chenyue Zhang, Ashleigh R Sharman, Julie Carlton, Ruijin Tang, Nicole M Rankin

**Affiliations:** 1 School of Public Health Faculty of Medicine and Health The University of Sydney Sydney Australia

**Keywords:** lung cancer screening, communication, recommendation, lung cancer, cancer, cross-sectional study, cancer screening, screening program, screening

## Abstract

**Background:**

Lung cancer is the leading cause of cancer death worldwide. The US Preventive Services Task Force (USPSTF) updated recommendations for lung cancer screening in 2021, adjusting the age of screening to 50 years (from 55 years) and reducing the number of pack-years used to estimate total firsthand cigarette smoke exposure to 20 (from 30). With many individuals using the internet to find health care information, it is important to understand what information is available for individuals contemplating lung cancer screening.

**Objective:**

This study aimed to assess the eligibility criteria and information available on lung cancer screening program websites for both health professionals and potential screening participants.

**Methods:**

A descriptive cross-sectional analysis of 151 lung cancer screening program websites of academic (n=76) and community medical centers (n=75) in the United States with information for health professionals and potential screening participants was conducted in March 2021. Presentation of eligibility criteria for potential screening participants and presence of information available specific to health professionals about lung cancer screening were the primary outcomes. Secondary outcomes included presentation of information about cost and smoking cessation, inclusion of an online risk assessment tool, mention of any clinical guidelines, and use of multimedia to present information.

**Results:**

Eligibility criteria for lung cancer screening was included in nearly all 151 websites (n=142, 94%), as well as age range (n=139, 92.1%) and smoking history (n=141, 93.4%). Age was only consistent with the latest recommendations in 14.5% (n=22) of websites, and no websites had updated smoking history. Half the websites (n=76, 50.3%) mentioned screening costs as related to the type of insurance held. A total of 23 (15.2%) websites featured an online assessment tool to determine eligibility. The same proportion (n=23, 15.2%) hosted information specifically for health professionals. In total, 44 (29.1%) websites referred to smoking cessation, and 46 (30.5%) websites used multimedia to present information, such as short videos or podcasts.

**Conclusions:**

Most websites of US lung cancer screening programs provide information about eligibility criteria, but this is not consistent and has not been updated across all websites following the latest USPSTF recommendations. Online resources require updating to present standardized information that is accessible for all.

## Introduction

The National Lung Screening Trial (NLST) demonstrated that annual low-dose computed tomography (LDCT) screening over 3 years can reduce lung cancer mortality in specific high-risk groups by 20% [1]. These findings were reinforced by results from the NELSON (Nederlands–Leuvens Longkanker Screenings Onderzoek) trial published in 2020, which, after a 10-year follow-up, demonstrated a reduction in lung cancer mortality to a similar magnitude of 24% [2].

Lung cancer screening involves identifying populations at high risk for the disease, with the aim to detect cancer at an early stage where curative treatment is available. The results of the NLST prompted the US Preventive Services Task Force (USPSTF) in December 2013 to recommend the implementation of LDCT screening [3]. The Level B recommendation was updated in March 2021, where age and smoking history were lowered to 50 years and 20 pack-years, respectively [4,5]. There was no change in the recommendation for the numbers of years quit for former smokers, which remained at 15 years.

Despite the implementation of lung cancer screening in the United States, screening uptake, according to the National Health Interview Survey in 2015, was estimated to be less than 4% of the 6.8 million American adults who meet the USPSTF screening criteria [6]. Screening uptake varies across US states, as demonstrated by self-reported data from the Behavioral Risk Factor Surveillance System in 2017, which showed uptake as high as 19.2% in Florida but lower uptake in Nevada (6.9%) and Georgia (11%) [7]. The combined uptake across these states was 16.3%.

A lack of awareness of screening, in both potential screening participants and health professionals, has been shown to be a challenge associated with implementing lung cancer screening [8]. Accessible and comprehensive information to address the information needs of potential screening participants may be important to promote a greater understanding of LDCT screening [9]. Potential screening participants may feel confused or anxious about the screening process, fear a cancer diagnosis or social stigma, and have cost concerns [10]; hence, they may seek answers from their family physician. Many health professionals discuss eligibility for lung cancer screening with potential screening participants according to the guidelines but often cannot achieve this equitably due to short consultation times [11]. Therefore, many potential screening participants turn to the internet for more health information, with the Health Information National Trends Survey showing that the internet is the first place people go to for health and medical information [12].

Use of the internet as a unique tool to facilitate interaction between health care providers and patients appears to be growing, and internet-based resources have been shown to increase participation in lung cancer screening [13]. It is important to understand whether potential screening participants are able to access accurate and reliable information and whether this information is consistent with current guidelines. Many US medical centers have created websites that are both academic and community focused and contain health and wellness program information, such as information about lung cancer screening. These sites may be the first or primary source of information about lung cancer screening for both potential screening participants and health professionals, and may affect their judgment on screening eligibility, how to navigate steps prior to screening, and, ultimately, uptake of screening.

A previous review examined these websites for benefits, harms, and recommended next steps for eligible individuals [14]. Given these websites are from academic and community-based lung cancer screening–designated centers in the United States and may be the first source of eligibility criteria for potential screening participants, our team aimed to assess whether eligibility criteria for potential screening participants were up-to-date on these websites following the latest updates to the USPSTF recommendation. As these websites may also be sources of information for health professionals, we wanted to investigate whether these websites contain any information to directly inform health professionals about lung cancer screening.

## Methods

### Procedure

The research team contacted the authors of a previously published article (Clark et al [14]) and obtained the list of 162 lung cancer screening program websites of academic centers (n=81) and state-matched community medical centers (n=81). Further detail on how the websites were selected is provided elsewhere [14].

Three team members each familiarized themselves with the content of 10 randomly selected websites. The team developed a data extraction tool to record eligibility criteria and other eligibility criteria (eg, family history, comorbidities) and whether there was information specific to health professionals (eg, link to an external website, a separate tab available on the website). We also recorded whether the websites mentioned any clinical guidelines (eg, USPSTF), included an online risk assessment tool, and gave any specific information about the cost of screening, whether smoking cessation advice was included, and whether there were any multimedia included on the websites.

The 162 websites were equally divided between 3 members of the team for data extraction. Using the data extraction sheet, we recorded whether each website was accessible, presented the above information or not, and details about what was included. Uncertainties about information were discussed, and decisions were resolved by the whole team. Each website took between 8 to 10 minutes to analyze and record the content into the checklist, with the checklist items iteratively updated during the process to reflect smoking cessation, specific cost, and use of multimedia.

Another member of the research team verified and updated the data extracted from all websites in March 2021, resolving any conflicts. A total of 11 websites were inaccessible due to main site errors. Where website pages were found to no longer be accessible, the institution homepage was accessed and the term “lung cancer screening” was entered into the search bar. Updated pages were then used for analysis. Where the original URL redirected to another website, the new page was used for analysis. All new web links were recorded.

### Analysis

Descriptive analysis was used to evaluate the frequencies of the information reported across the websites. Statistical analysis was carried out using Microsoft Excel (Microsoft Corp).

### Ethics Approval

Ethics approval was not required as the websites are in the public domain, and no human participants were involved.

## Results

### Details of Websites

Of the 162 websites, 11 were no longer accessible. Of the remaining 151 websites, (academic websites: n=76; community websites: n=75; Multimedia Appendix 1), 13 academic websites and 26 community websites had URL changes, largely because lung cancer screening information had been mapped to a different section of the website or a new website was built or refreshed (see an example in Multimedia Appendix 2). Therefore, of the 151 included websites, 39 website URLs were different from the original URLs reported by Clark and colleagues [14].

### Eligibility Criteria

#### Age

Overall, 62.9% (95/151) of websites mentioned at least one professional guideline for lung cancer screening eligibility (Table 1). The standard age ranges reported across the 151 websites varied greatly. The 3 most reported eligible age groups were 55 to 77 years (n=66, 43.7%), 55 to 80 years (n=40, 26.5%), and 55 to 74 years (n=18, 11.9%); 17.2% (n=26) of websites mentioned more than one age group. Age was consistent with the latest USPSTF recommendations (≥50 years) in 22 (14.5%) websites but was mentioned specifically (ie, age of 50-80 years) in only 7 (4.6%) websites.

**Table 1:** Information about eligibility criteria on the lung cancer screening websites of academic and community centers in the United States.

Eligibility criteria			Academic center (n=76), n		Community center (n=75), n		Total (N=151), n (%)
**Age range (years)**							
	≥50	9		3		12 (7.9)	
	>50	0		3		3 (2.0)	
	≥55	2		5		7 (4.6)	
	>55	1		0		1 (0.7)	
	50-74	1		2		3 (2.0)	
	50-77	0		1		1 (0.7)	
	50-80	6		1		7 (4.6)	
	55-70	0		1		1 (0.7)	
	55-74	11		7		18 (11.9)	
	55-77	34		32		66 (43.7)	
	55-78	0		1		1 (0.7)	
	55-79	2		1		3 (2.0)	
	55-80	20		20		40 (26.5)	
	55-88	1		0		1 (0.7)	
	Not mentioned	4		8		12 (7.9)	
**Smoking history**							
	Is a current smoker or has quit smoking within the last 15 years	73		68		141 (93.4)	
	Has a smoking history of at least 30 pack-years^a^	61		57		118 (78.1)	
	Not mentioned	3		7		10 (6.6)	
**Guidelines mentioned**							
	USPSTF^b^/NCCN^c^/ACR^d^/ACS^e^/others^f^	31		19		50 (33.1)	
	National Lung Screening Trial (National Cancer Institute)	23		10		33 (21.9)	
	Medicare/Medicaid/private insurance plans	27		23		50 (33.1)	
	Not mentioned	23		33		56 (37.1)	
**Other criteria**							
	Family history	10		11		21 (13.9)	
	Occupational or environmental exposure	12		13		25 (16.6)	
	No signs or symptoms of lung cancer, asymptomatic	17		23		40 (26.5)	
	Not mentioned	25		37		62 (41.1)	

^a^Pack-years: packs per day multiplied by the number of years a person has smoked (meaning 1 pack a day for 30 years, 2 packs a day for 15 years, etc).

^b^USPSTF: US Preventive Services Task Force.

^c^NCCN: National Comprehensive Cancer Network.

^d^ACR: American College of Radiology.

^e^ACS: American Cancer Society.

^f^Others mentioned only once include the American Thoracic Society, the American Society of Clinical Oncology, the American Lung Association, the Centers for Disease Control and Prevention, and the American Society of Clinical Oncology.

#### Smoking History

**Figure 1:**
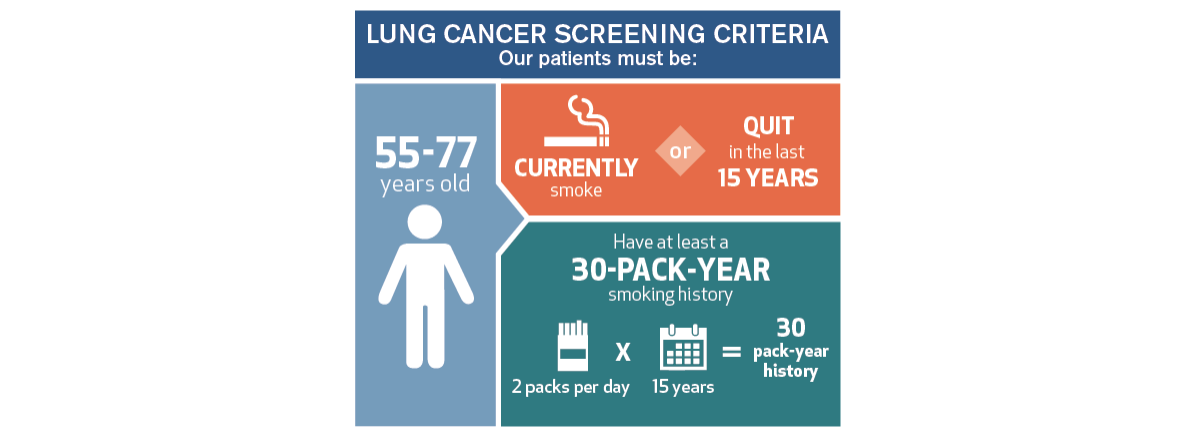
Example eligibility criteria from Houston Methodist [15].

Most websites (n=141, 93.4%) listed the eligibility criteria of smoking history, while 78.1% (n=118) detailed information on those who have a 30 pack-year smoking history (see example in Figure 1 [15]). None of the websites had updated pack-year smoking history in line with the latest recommendations. In addition to the eligibility criteria listed in the guidelines, the most frequently mentioned other eligibility criteria were asymptomatic status (n=40, 26.5%), occupational or environmental exposure (n=25, 16.6%), and family history (n=21, 13.9%).

### Eligibility Criteria Using an Online Assessment Tool

A total of 23 (15.2%) websites promoted the use of an online assessment tool to determine eligibility for screening; 20 (13.2%) were related to risk and 3 (2%) were related to pack-year calculation only.

### Information Targeted at Health Professionals

Among the 151 websites, 15.2% (n=23) listed information specifically for health professionals, with academic websites accounting for 26.3% (20/76) and community websites making up 4% (3/75). The most common resources were links to refer patients to treatment centers and PDF downloads, including order forms, patient booklets, shared decision aid guides, and posters.

### Cost of Lung Cancer Screening

A total of 76 (50.3%) websites referred to the cost of lung cancer screening. Of these, 73.7% (n=56) mentioned that cost would be related to the type of insurance coverage held (ie, Medicare, Medicaid, or private insurers); 5.3% (n=4) mentioned self-pay cost only, ranging from US $99 to US $350; 17.1% (n=13) mentioned insurance coverage and self-pay cost, ranging from US $99 to US $361; 1.3% (n=1) mentioned insurance coverage and available scholarships (eg, Lung Cancer Screening Scholarships, funded by the McLeod Foundation’s McLeod Men’s group and McLeod Angels); and 2.6% (n=2) mentioned free screening where criteria were met (eg, a free annual LDCT lung cancer screening for those considered high risk and meeting Medicare’s screening criteria).

### Smoking Cessation Programs

A total of 44 (29.1%) websites referred to smoking cessation. Of these, 34.1% (n=15) mentioned that smoking cessation information or counseling was included in the screening program; 52.3% (n=23) provided information to access an in-house smoking cessation program; 20.5% (n=9) provided information for local, city, or state-based smoking cessation programs; 11.4% (n=5) provided information for national smoking cessation programs; and 2.3% (n=1) made a recommendation to enter a smoking cessation program but did not provide any further resources.

### Multimedia Targeted at Participants

Multimedia formats were used in 30.5% (n=46) of websites to present information on topics such as promoting the benefits and harms of lung cancer screening and explaining the process of screening. Of these 46 websites, 67.4% (n=31) presented short video clips, 17.4% (n=8) presented patient testimony, 10.9% (n=5) presented podcasts, and 8.7% (n=4) presented infographics.

### Promoted Associations and Registrations

A total of 49 (32.5%) websites referred to or displayed the logo of one or more relevant associations or membership registration. Over a quarter of the websites (40/151, 26.5%) listed were an American College of Radiology Lung Cancer Screening Center; 8.6 (n=13) were a Lung Cancer Alliance Screening Center of Excellence; 7.9% (n=12) were a GO2 Foundation for Lung Cancer Center of Excellence; 2% (n=3) were a National Cancer Institute–designated Comprehensive Cancer Center; and 1.3% (n=2) were a Commission on Cancer Accredited Program.

## Discussion

### Principal Findings

Our findings demonstrate that information was not standardized across websites about lung cancer screening, with the majority being out of date with the latest USPSTF recommendations regarding the revised eligibility criteria of a younger starting age and a reduced smoking history. About two-thirds of websites that referred to professional society guidelines were consistent in their recommendations about eligibility. The potential costs of screening and smoking cessation programs were less often reported on websites. Given the poor uptake of lung cancer screening across the United States, it is important to ensure potential screening participants can access accurate and sufficiently detailed information to determine and understand their eligibility.

#### General Population

The internet is a central source of health information that can empower patients, promote knowledge, and support decision-making [16]. When developing these community-facing websites, all the required information should aim to be in a format that is accessible to all language and literacy groups [17] and follow plain English guidelines as endorsed by the World Health Organization [18]. This is particularly important given the socioeconomic disparities known to exist among those who will be eligible for lung cancer screening [19]. For knowledge transfer and support in decision-making to occur, the information needs to be accurate and should be updated regularly by the institution, but the responsibility of evaluating health information found online lies with the consumer [20]. For website creators to maintain the accuracy of the information provided, this would require a standardized assessment tool such as the Health Sector Website Assessment Index, which assesses content, services, community interaction, and technological features [21]. Although this index is not suitable for this context, a multi-indicator tool that is easy to assess websites could be developed for regular auditing of websites containing health information to ensure the information stays up-to-date.

Previous research has found that health professionals have low awareness of eligibility criteria for lung cancer screening, showing that less than 50% are able to correctly answer the eligibility criteria for lung cancer screening [22,23]. These findings suggest that the conflicting information provided by these lung cancer screening program websites may confuse both potential screening participants and health professionals. For example, the USPSTF recommendations list the upper age limit for screening as 80 years old [24] whereas the upper age limit covered by the Centers for Medicare and Medicaid Services is 77 years old [25] and that listed on the websites of the NLST [1] and the American Cancer Society [26] is 74 years old. Variations in age given across the websites were explained to be due to differences in insurance coverage, risk factors, and recommended guidelines.

Having an interactive online tool that combines the age and smoking eligibility criteria was utilized in only a few of these websites, but provides a tangible tool for potential participants of lung cancer screening to determine their eligibility quickly. Online decision support tools have been shown to be efficiently implemented in breast cancer risk assessment, as well as in facilitating shared decision-making [27]. Providing these online tools can empower potential participants to determine their eligibility prior to approaching their family physician. Similarly, the use of multimedia tools on websites can aid in the understanding of potential participants, with incorporation of multimedia resources into the informed consent process shown to be preferred by culturally and linguistically diverse patients [28]. Providing interactive videos and tools on these websites may improve the understanding of potential participants and consequently improve participation in lung cancer screening.

In addition, this study found that smoking history and time to quit smoking are also prevalent on most websites as screening eligibility criteria. Despite this, only a third referred to smoking cessation resources. As lung cancer screening may provide an excellent opportunity to approach smoking cessation and act as a “teachable moment” [29,30], providing smoking cessation resources on these websites presents a unique opportunity to reach those at high risk of lung cancer who may be motivated to quit [31].

#### Health Professionals

Although most websites list recommended next steps for potential screening participants to take, few health professionals are given specific information to help guide these consultations and direct potential screening participants to a local health care team. For health professionals, the challenges generally include lack of awareness of eligibility standards and insurance coverage, difficulty in identifying eligible patients, insufficient time [32] or knowledge to make joint decisions, and the need for management guidance on lung cancer screening results and the balance between benefits and harms [10].

Of all the websites evaluated in this study, only 1 in 6 highlighted the important role that health professionals play in encouraging potential screening participants to consider participation. This study examined the content of lung cancer screening program websites, which may be the main source of information for many health professionals and potential screening participants. These sites provide an opportunity to fully cover eligibility criteria, screening costs, and recommended next steps. Providing this information may complement the shared decision-making process that occurs prior to screening, which aims to ensure patients make an informed choice about whether to undergo screening, and can improve outcomes [33].

Although these lung cancer screening program websites are not responsible for fully providing information recommended by the guidelines for shared decision-making, they can provide helpful advice for eligible individuals and advise them on the next steps when considering screening.

### Limitations

This study has some limitations. It is possible that our content review of each website may have missed or misinterpreted some content, but by having a structured data extraction tool, as well as having 3 researchers randomly assigned to review the websites and a fourth who checked for accuracy and updates, we consider this limitation to be minimized. We limited the website review to focus on the key components of eligibility, but we may have missed other details that could influence the patient’s decision-making process such as distance to travel to a screening facility or convenience of when screening was available [8]. In addition, although online health information is now a main resource for patients and health professionals, we had no access to information about how often these websites are visited or what role they play in their decision-making process.

### Conclusion

The study found that the information provided to health professionals and potential screening participants on the lung cancer screening program websites is not standardized or up to date with the latest USPSTF recommendations. Few websites mentioned the information needed for health professionals to facilitate shared decision-making. Considering the wide impact and potential low cost of using internet strategies to obtain health information, these findings can be used to inform the development of online resources for potential screening participants and health professionals, with the focus on presenting standardized information that is accessible to all literacy levels. Future qualitative research with potential screening participants and health professionals exploring their use of websites for lung cancer screening information would be beneficial.

## Appendix

Multimedia Appendix 1 Websites included in the study.

Multimedia Appendix 2 Examples of website changes.

## Abbreviations

LDCT: low-dose computed tomography
NELSON: Nederlands–Leuvens Longkanker Screenings Onderzoek
NLST: National Lung Screening Trial
USPSTF: US Preventive Services Task Force
