# Supplementary material for: Assessing Information Available for Health Professionals and Potential Participants on Lung Cancer Screening Program Websites: Cross-sectional Study
Source: JMIR Cancer. 2022 Aug 30;8(3):e34264. doi: 10.2196/34264 (PMC9472061; doi:10.2196/34264)
Supplement: Multimedia Appendix 1 [file cancer_v8i3e34264_app1.docx]

| **Appendix: Websites included in the study** | | |
| --- | --- | --- |
|  | **Institution** | **Website Link** |
|  | **ACADEMIC** | |
| 1 | University of Alabama School of Medicine | <https://www.uab.edu/medicine/radiology/patient-care/lung-cancer-ct-screening> |
| 2 | University of Arkansas for Medical Sciences/UAMS College of Medicine | <https://cancer.uams.edu/patients-family/prevention/lung-cancer-screening/> |
| 3 | Keck School of Medicine of University of Southern California | <https://cancer.keckmedicine.org/lungscreening/> |
| 4 | Loma Linda University School of Medicine | https://lluh.org/cancer-center/cancer-programs/lung-cancer-care/faq-lung-cancer-screening |
| 5 | Stanford University School of Medicine | <https://stanfordhealthcare.org/medical-tests/c/ct-lung-cancer-screening.html> |
| 6 | University of California, Davis School of Medicine | <https://www.ucdmc.ucdavis.edu/surgery/specialties/cardio/lung_cancer_screen.html> |
| 7 | University of California, Irvine School of Medicine | <http://www.ucihealth.org/medical-services/lung-cancer/lung-cancer-screening> |
| 8 | Rush Medical College | <https://www.rush.edu/services/test-treatment/lung-cancer-screening> |
| 9 | University of Chicago Pritzker School of Medicine | <https://www.uchicagomedicine.org/conditions-services/cancer/lung-cancer/screening> |
| 10 | University of Illinois College of Medicine | <https://hospital.uillinois.edu/primary-and-specialty-care/cancer-services-at-ui-health/cancer-programs/lung-cancer/lung-cancer-screening> |
| 11 | Indiana University School of Medicine | <https://iuhealth.org/find-medical-services/lung-cancer> |
| 12 | University of Kansas School of Medicine | <https://www.kucancercenter.org/cancer/cancer-types/lung-cancer/lung-cancer-diagnosis-screening/low-dose-ct> |
| 13 | University of Kentucky College of Medicine | <https://ukhealthcare.uky.edu/markey-cancer-center/patient-care/cancer-screening-program/lung> |
| 14 | University of Louisville School of Medicine | <https://uoflbrowncancercenter.org/lung-screening> |
| 15 | Tulane University School of Medicine | <https://tulanehealthcare.com/service/lung-cancer-screening> |
| 16 | Johns Hopkins University School of Medicine | <https://www.hopkinsmedicine.org/healthlibrary/conditions/adult/respiratory_disorders/respiratory_disorders_22,LungCancerScreening> |
| 17 | University of Maryland School of Medicine | <https://www.umms.org/bwmc/health-services/lung-screening-program> |
| 18 | Boston University School of Medicine | <https://www.bmc.org/lung-cancer-screening-and-lung-nodule-evaluation-program> |
| 19 | University of Massachusetts Medical School | <https://www.umassmemorialhealthcare.org/healthalliance-clinton-hospital/ct-lung-cancer-screening> |
| 20 | University of Michigan Medical School | <https://www.uofmhealth.org/conditions-treatments/pulmonary/lung-cancer-screening-clinic> |
| 21 | Mayo Clinic College of Medicine | <https://www.mayoclinic.org/tests-procedures/lung-cancer-screening/about/pac-20385024> |
| 22 | University of Minnesota Medical School | <https://www.mhealth.org/care/treatments/lung-cancer-screening-adult> |
| 23 | University of Mississippi School of Medicine | <https://www.umc.edu/Healthcare/Cancer/Cancer_Screening/Lung%20Cancer%20Screening%20and%20Diagnosis.html> |
| 24 | Washington University School of Medicine | <https://siteman.wustl.edu/treatment/cancer-types/lung-cancer/lungcancerscreening/> |
| 25 | Creighton University School of Medicine | <https://www.chihealth.com/en/services/cancer-care/lung-health-centers.html> |
| 26 | University of Nebraska College of Medicine | <https://www.nebraskamed.com/cancer/lung-screening> |
| 27 | Dartmouth College Geisel School of Medicine | <https://cancer.dartmouth.edu/lung-thoracic/lung-cancer-screening> |
| 28 | Georgetown University School of Medicine | <https://www.medstargeorgetown.org/our-services/cancer-care/lung-cancer/conditions/lung-cancer-risk-factors-and-screening/> |
| 29 | University of Florida College of Medicine | <https://ufhealth.org/uf-health-lung-cancer-program/lung-cancer-screening-information> |
| 30 | University of Miami Leonard M. Miller School of Medicine | <https://umiamihealth.org/sylvester-comprehensive-cancer-center/treatments-and-services/lung-and-chest-cancer/lung-cancer-screening> |
| 31 | Emory University School of Medicine | <https://www.emoryhealthcare.org/radiology/diagnosis/ct-scan-lungs.html> |
| 32 | Medical College of Georgia at Augusta University | <https://www.augustahealth.org/cancer-care/lung-cancer> |
| 33 | Loyola University Chicago Stritch School of Medicine | <https://www.loyolamedicine.org/cancer/lung-cancer-screening-guidelines> |
| 34 | Northwestern University Feinberg School of Medicine | <https://www.nm.org/conditions-and-care-areas/cancer-care/lung-cancer-care/lung-cancer-care-in-the-northern-suburbs/screening-lung-ct-scan> |
| 35 | Drexel University College of Medicine | <https://towerhealth.org/screenings> |
| 36 | Pennsylvania State University College of Medicine | <https://www.pennstatehealth.org/services-treatments/lung-cancer-screening> |
| 37 | Perelman School of Medicine at the University of Pennsylvania | <https://www.pennmedicine.org/cancer/types-of-cancer/lung-cancer/small-cell-lung-cancer/lung-cancer-screening> |
| 38 | Sidney Kimmel Medical College at Thomas Jefferson University | <https://hospitals.jefferson.edu/departments-and-services/lung-cancer-screening-program.html> |
| 39 | Temple University School of Medicine | <https://lung.templehealth.org/patient-care/centers-programs/lung-cancer-screening-program> |
| 40 | University of Pittsburgh School of Medicine | <https://www.upmc.com/services/south-central-pa/lung/surgery/thoracic/lung-cancer/screening> |
| 41 | Medical University of South Carolina College of Medicine | <https://www.hollingscancercenter.org/patient-care/cancer-types/lung/screening.html> |
| 42 | University of Tennessee College of Medicine | <https://www.utmedicalcenter.org/medical-care/prevention-screening/low-dose-ct-lung-cancer-screening/> |
| 43 | Vanderbilt University School of Medicine | https://www.vanderbilthealth.com/program/lung-cancer-screening |
| 44 | Baylor College of Medicine | <https://www.bswhealth.com/specialties/imaging-and-radiology/Pages/lung-cancer-screening-program.aspx> |
| 45 | University of Texas Southwestern Medical School at Dallas | <https://utswmed.org/conditions-treatments/lung-cancer/ct-screening-lung-cancer/> |
| 46 | University of Virginia School of Medicine | <https://uvahealth.com/services/lung-cancer/how-to-get-a-screening> |
| 47 | VCU School of Medicine, Medical College of Virginia Health Sciences Division | <https://www.massey.vcu.edu/patient-care/prevention-screening/lung-cancer-screening/> |
| 48 | University of Washington School of Medicine | <https://www.uwmedicine.org/lung-cancer/> |
| 49 | Medical College of Wisconsin | <https://www.froedtert.com/lung-cancer/screening> |
| 50 | University of Wisconsin School of Medicine and Public Health | https://www.uwhealth.org/conditions/lung-cancer |
| 51 | David Geffen School of Medicine at UCLA | <https://www.uclahealth.org/lungcancer/lung-screening-clinic> |
| 52 | University of California, San Diego School of Medicine | <https://health.ucsd.edu/specialties/cancer/programs/lung/Pages/CT-screening.aspx> |
| 53 | UCSF School of Medicine | <https://www.ucsfhealth.org/programs/lung_cancer_screening_program/> |
| 54 | University of Colorado School of Medicine | <https://www.uchealth.org/services/cancer-care/treatment/lung-and-thoracic-cancer/lung-cancer-screening-program/> |
| 55 | University of Connecticut School of Medicine | <https://health.uconn.edu/cancer/patient-services/prevention-screening/lung-cancer-screening/> |
| 56 | Yale School of Medicine | <https://www.yalecancercenter.org/patient/programs/thoracic/specialties/screening/index.aspx> |
| 57 | George Washington University Medical School | <https://www.gwhospital.com/conditions-services/cancer-center/lung-cancer/lung-cancer-screening> |
| 58 | Cooper Medical School of Rowan University | <https://www.cooperhealth.org/services/high-risk-lung-cancer-screening-program> |
| 59 | Rutgers Robert Wood Johnson Medical School | <http://www.cinj.org/patient-care/lung-cancer-screening-program> |
| 60 | University of New Mexico School of Medicine | <http://cancer.unm.edu/cancer/cancer-info/testing-overview/screening/lung-cancer-screening/> |
| 61 | Albert Einstein College of Medicine | <https://www.einstein.edu/cancer/treatments/lung-cancer> |
| 62 | Columbia University Roy and Diana Vagelos College of Physicians and Surgeons | <http://www.cumc.columbia.edu/pulmonary/clinical-centers/lung-cancer-screening-program> |
| 63 | Icahn School of Medicine at Mount Sinai | <https://www.mountsinai.org/care/radiology/services/lung-screening> |
| 64 | New York University School of Medicine | <https://nyulangone.org/locations/lung-cancer-screening-program> |
| 65 | Stony Brook University School of Medicine | <https://cancer.stonybrookmedicine.edu/patients/lung-cancer-screening-program> |
| 66 | State University of New York Upstate Medical University | <http://www.upstate.edu/cancer/prevention/screening/lung-program.php> |
| 67 | Jacobs School of Medicine and Biomedical Sciences, University at Buffalo | <https://www.roswellpark.org/cancer/lung/prevention-screening/lung-screening-program> |
| 68 | University of Rochester School of Medicine and Dentistry | <https://www.urmc.rochester.edu/encyclopedia/content.aspx?contenttypeid=34&contentid=16337-1> |
| 69 | Weill Cornell Medical College | <https://wcinyp.org/patients/radiology-consultation-services/lung-cancer-screening> |
| 70 | Duke University School of Medicine | <https://radiology.duke.edu/patient-care/specialized-services/lung-cancer-screening/> |
| 71 | University of North Carolina School of Medicine | <https://unclineberger.org/patientcare/programs/lung-cancer-screening-clinic> |
| 72 | Wake Forest School of Medicine | <https://www.wakehealth.edu/Diagnostic-Tools/Low-Dose-CT-Lung-Screening> |
| 73 | Cleveland Clinic Lerner College of Medicine | <https://my.clevelandclinic.org/health/treatments/15031-lung-cancer-screening-program-> |
| 74 | The Ohio State University College of Medicine | <https://cancer.osu.edu/cancer-specialties/cancer-care-and-treatment/lung-cancers/lung-cancer-screening-clinic> |
| 75 | University of Cincinnati College of Medicine | <https://uchealth.com/services/lung-cancer/> |
| 76 | Oregon Health & Science University School of Medicine | <https://www.ohsu.edu/xd/education/schools/school-of-medicine/departments/clinical-departments/diagnostic-radiology/patient-imaging-services/lung-cancer-screening-program.cfm> |
|  | **COMMUNITY** | |
| 77 | Adirondack Medical Center - CT | <https://lakehealth.org/service/cancer-care/cancer-screenings/lung-cancer-screenings/> |
| 78 | Adventist Bolingbrook Hospital | <https://www.amitahealth.org/find-a-service/medical-imaging/computed-tomography-ct-lung-cancer-screening> |
| 79 | Advocate BroMenn Medical Center | <https://www.advocatehealth.com/health-services/cancer-institute/cancers-we-treat/lung-cancer/screening-diagnosis> |
| 80 | Alhambra Hospital Medical Center | <https://www.stlukes-stl.com/services/oncology/lung-cancer-screening-program.html> |
| 81 | Allegheny General Hospital | <https://www.ahn.org/services/cancer/types/lung/screening> |
| 82 | Angel Medical Center | <https://www.crozerhealth.org/services/lung-respiratory-care/lung-screening-program/> |
| 83 | Arnot-Ogden Medical Center | <http://healthlibrary.integrisok.com/RelatedItems/34,16337-1> |
| 84 | Atchison Hospital | <https://amberwellhealth.org/service/radiology/> |
| 85 | Atlantic General Hospital | <http://www.atlanticgeneral.org/Our-Services/Imaging-Radiology/Low-Dose-CT-Lung-Cancer-Screenings.aspx> |
| 86 | Augusta Health | <https://www.augustahealth.com/cancer-center/lung-cancer-screening> |
| 87 | Aurora Lakeland Medical Center | <https://www.aurorahealthcare.org/services/cancer/lung-cancer#Screenings> |
| 88 | Baptist Health Medical Conway | <https://www.baptist-health.com/page/low-dose-ct-lung-screening> |
| 89 | Berkshire Medical Center | <http://www.berkshirehealthsystems.org/lung-cancer-screening> |
| 90 | Brandywine Hospital- CT | <https://towerhealth.org/screenings> |
| 91 | Cannon Memorial Hospital | <http://anmedhealth.org/Services/Cancer-Care/Screening-Prevention/Lung-Cancer-Screening> |
| 92 | Cascade Medical Center | <https://cascademedical.org/services/diagnostic-imaging> |
| 93 | Christiana Health Services - Wilmington Hospital | <https://christianacare.org/health/healthtools/lunghealthandscreening/> |
| 94 | Christus Santa Rosa Hospital - New Braunfels | <https://www.christushealth.org/santa-rosa/new-braunfels/services-treatments/imaging--radiology/ct-lung-screening> |
| 95 | Community Hospital | <https://www.ecommunity.com/services/cancer-care/lung-cancer/lung-cancer-screening> |
| 96 | Crozer Chester Medical Center - Springfield Division | <https://www.mhs.net/services/cancer/types/lung/screening> |
| 97 | Ellis Hospital | <https://missionhealth.org/services-treatments/cancer-care/low-dose-lung-screening/> |
| 98 | Essentia Health St. Josephs Medical Center | <https://www.essentiahealth.org/services/pulmonary-medicine/lung-cancer-screening/> |
| 99 | Fairview Hospital | <http://www.griffinhealth.org/radiology/diagnostic-imaging/computerized-tomography/low-dose-ct-lung-cancer-screening> |
| 100 | Frick Hospital/ Excela Health | <https://www.excelahealth.org/services/lung-care-advanced-lung-center/lung-cancer-screening-appointments> |
| 101 | Griffin Hospital | <https://lourdesrmc.com/services/imaging-services/lung-cancer-screening-ct-scan> |
| 102 | Gundersen Health System | <https://www.gundersenhealth.org/services/cancer/lung-cancer/screening/> |
| 103 | Hackettstown Medical Center | <https://www.atlantichealth.org/conditions-treatments/pulmonary-services/lung-cancer-screening-program.html> |
| 104 | Hardin Memorial Hospital | <https://www.hmh.net/low-dose-ct-scan/> |
| 105 | Henrico Doctors Hospital | <https://www.adirondackhealth.org/services-conditions/medical-imaging> |
| 106 | Holy Cross Hospital | <https://www.holy-cross.com/lung-cancer-screening-low-dose-ct-99> |
| 107 | Houston Methodist Sugar Land Hospital | <https://www.houstonmethodist.org/cancer/lung-cancer/screening/> |
| 108 | Inova Alexandria Hospital | <https://www.tbh.org/lung-cancer-screening-program> |
| 109 | Inspira Medical Center-Vineland | <https://www.inspirahealthnetwork.org/services-treatments/cancer-care/cancer-screenings/lung-cancer-screenings> |
| 110 | INTEGRIS Baptist Medical Center, Inc. d/b/a | <https://henricodoctors.com/blog/entry/what-you-need-to-know-about-lung-cancer-screening> |
| 111 | J. T. Mather Memorial Hospital | <http://www.northernhospital.com/hospital-services/imaging-services/lungscreening> |
| 112 | Lake Health West Medical Center | <https://healthonecares.com/specialties/lung-cancer?location=healthone-cares> |
| 113 | Leconte Medical Centre | <https://www.lecontemedicalcenter.com/lung-cancer-screening-expectations/> |
| 114 | Lehigh Valley Hospital-Pocono Bartonsville Healthcare Center | <http://www.poconohealthsystem.org/LDCT> |
| 115 | Lexington Medical Center | <http://www.lexmed.com/medical-services/cancer-center/early-detection/lung-cancer-screening> |
| 116 | Maimonides Medical Center | <https://www.saintalphonsus.org/lung-screening> |
| 117 | McCullough-Hyde Memorial Hospital | <https://middlesexhealth.org/cancer-center/cancer-types/lung-cancer/lung-cancer-screening> |
| 118 | McLeod Regional Medical Center | <https://www.mcleodhealth.org/services/care/cancer-center/lung-screening/> |
| 119 | Memorial Regional Hospital | <https://www.rochesterregional.org/services/pulmonary-critical-care/lung-cancer-screening-program> |
| 120 | Mercy Medical Center | <https://www.mercyone.org/northiowa/find-a-service-or-specialty/cancer-care/lung-cancer-screening> |
| 121 | Methodist Women's Hospital | <https://bestcare.org/specialties/lung-and-thoracic-oncology> |
| 122 | Middlesex Hospital- Marlborough Medical Center | <https://www.matherhospital.org/care-treatment/imaging-services-radiology/lung-cancer-screening/> |
| 123 | OSF Saint Francis Medical Center | <https://www.osfhealthcare.org/pulmonology/services/testing/low-dose-ct/> |
| 124 | Our Lady of Lourdes Hospital | <https://www.nhrmc.org/services/radiology/imaging-tests-procedures/lung> |
| 125 | Overlake Hospital Medical Center | <http://healthlibrary.overlakehospital.org/Conditions/Cancer/Specific/Lung/Prevention/34,16337-1> |
| 126 | Parkridge Medical Center | <https://parkridgehealth.com/service/lung-cancer-screening-in-chattanooga> |
| 127 | Pella Regional Health Center | <https://www.pellahealth.org/services/medical-imaging/lung-cancer-screening/> |
| 128 | Pender Memorial Hospital | <https://www.providence.org/treatments/lung-cancer-screening> |
| 129 | Providence Holy Cross Health Center-Santa Clarita | <https://www.providence.org/treatments/lung-cancer-screening> |
| 130 | Providence Hospital | <https://healthcare.ascension.org/Specialty-Care/Cancer/Lung-Cancer-Screening-at-Providence> |
| 131 | Providence Little Company of Mary Medical Center San Pedro | <https://www.tmcaz.com/lungscreen> |
| 132 | Providence Saint Joseph Medical Center | <https://www.providence.org/treatments/lung-cancer-screening> |
| 133 | Queen of the Valley Medical Center | <https://www.providence.org/treatments/lung-cancer-screening> |
| 134 | Riverside Medical Center West Campus | <https://www.riversidehealthcare.org/services/cancer-institute/cancers-we-treat/lung-cancer/screening-and-diagnosis> |
| 135 | Rush Foundation Hospital | <https://www.rushhealthsystems.org/specialties-services/rush-imaging-center/ct-lung-cancer-screening/> |
| 136 | Saddleback Memorial Medical Center | <https://www.memorialcare.org/services/cancer-care/ct-scan-screening-early-lung-cancer-detection> |
| 137 | Saint Alphonsus Medical Center Baker City | <https://www.inova.org/our-services/lung-cancer-screening-program> |
| 138 | Southern New Hampshire Medical Center | <https://www.snhhealth.org/our-services/lung-cancer-screening> |
| 139 | St. John's Regional Medical Center | <https://www.dignityhealth.org/central-coast/locations/stjohnsregional/services/cancer-care/lung-screening-program-and-center-for-thoracic-oncology> |
| 140 | St. Joseph Mercy Livingston Hospital | <https://www.stjoeshealth.org/find-a-service-or-specialty/cancer-care/types-of-cancer/lung-cancer/lung-cancer-screening> |
| 141 | St. Luke's Roosevelt Hospital (St. Lukes Division) | <https://www.maimonidesmed.org/cancer-center/cancer-types-of-cancer/cancer-lung-cancer/our-preventive-diagnostic-services> |
| 142 | St. Tammany Parish Hospital | <https://www.sttammany.health/News/MBPOffersLowDoseCTLungScreenings> |
| 143 | The Brooklyn Hospital Center | <https://www.fairview.org/sitecore/content/Fairview/Home/Patient-Education/Articles/English/l/u/n/g/_/Lung_Cancer_Screening_Frequently_Asked_Questions_521737> |
| 144 | The Carle Foundation Hospital | <https://carle.org/Newsroom/Culture-Of-Quality/2020/11/Low-dose-screening-gives-smokers-early-insight-int> |
| 145 | The Medical Center of Aurora South Campus | [www.ellismedicine.org/cancer/lung-cancer-screening.aspx](http://www.ellismedicine.org/cancer/lung-cancer-screening.aspx) |
| 146 | Torrance Memorial Medical Center | <https://www.torrancememorial.org/Medical_Services/Cancer_Institute/Prevention_Screening_and_Diagnosis/Lung_Cancer_Screening.aspx> |
| 147 | Tucson Medical Center | <https://www.arnothealth.org/services/lung> |
| 148 | United Memorial Medical Center | <https://www.trihealth.com/institutes-and-services/trihealth-cancer-institute/types-of-cancer/why-trihealth-for-your-lung-cancer-treatment/lung-program/lung-cancer-screening-program> |
| 149 | Wellington Regional Medical Center | <https://www.wellingtonregional.com/services/comprehensive-lung-program> |
| 150 | Wellstar Windy Hill Hospital | <https://www.wellstar.org/education/pages/low-dose-ct-lung-cancer-screening.aspx> |
| 151 | Winchester Medical Center | <https://www.valleyhealthlink.com/Our-Services/Radiology-Medical-Imaging/Low-Dose-Lung-CT.aspx> |
