# Supplementary material for: Assessing Information Available for Health Professionals and Potential Participants on Lung Cancer Screening Program Websites: Cross-sectional Study
Source: JMIR Cancer. 2022 Aug 30;8(3):e34264. doi: 10.2196/34264 (PMC9472061; doi:10.2196/34264)
Supplement: Multimedia Appendix 2 [file cancer_v8i3e34264_app2.docx]

**Appendix: Examples of website changes.**

| **Community - Allegheny General Hospital**  Clark et a [14]: https://www.ahn.org/specialties/esophageal-and-lung-institute/free-risk-test  Current: https://www.ahn.org/services/cancer/types/lung/screening  **Academic - Pennsylvania State University College of Medicine**  Clark et al [14]: https://hmc.pennstatehealth.org/thoracic-surgery/lung-cancer-screening  Current: https://www.pennstatehealth.org/services-treatments/lung-cancer-screening |
| --- |
